# Gaining insights into a funding portfolio through publication tracking

**DOI:** 10.3310/nihropenres.14022.1

**Published:** 2025-07-22

**Authors:** Reetika Suri-Ogilvie, Sandra Hicks, Dominique Capostagno, Ashley Banks, Elena Ahmed, Kelly Makarona

**Affiliations:** 1 NIHR Infrastructure, National Institute of Health and Care Research, London, UK

**Keywords:** funding, portfolio, publications, citations, case studies, Research Excellence Framework

## Abstract

**Background:**

The National Institute for Health and Care Research (NIHR) is the UK’s biggest funder for health and social care research, funded by the Department of Health and Social Care (DHSC). The NIHR infrastructure provides research expertise, specialist facilities, a research delivery workforce and support services, all of which help to support and deliver the research we fund, and research funded by others. The NIHR is committed to maximising the impact of the research we support and fund ^1^ and therefore, it is crucial for the organisation to understand the mechanisms for the movement of research between these different pieces of translational research infrastructure and pathways to impact on the health and wealth of the nation. The aim of this article is to share our approach to developing an understanding of pathways to impact, enablers and barriers and lessons learnt.

**Methods:**

We used publications reported to us by our infrastructure as receiving infrastructure support and forward and backward citation analysis to trace infrastructure support for REF 2021 impact case studies and research that has had an impact on policy. We used these data to develop impact case studies for NIHR infrastructure.

**Results:**

Of the 6,361 REF impact case studies that are publicly available, the NIHR infrastructure has supported 327 of which 59 are supported by more than one scheme. Through our forward and backward citation analysis we have also developed impact case studies in the following NIHR priority areas:

- Reducing health inequalities
- Digital health
- Artificial intelligence
- Workforce resilience

**Conclusions:**

The use of forward and backward citation analysis can also help research funders to understand how research is moving between different parts of their funding portfolios, pathways to impact and any gaps and opportunities. However, this comes with some challenges which need mitigation.

## Plain English Summary

The National Institute of Health and Care research (NIHR) is funded by the Department of Health and Social Care. It funds a £600M/year portoflio of infrastructure which provided support and a delivery workforce for experimental and clinical research in England. NIHR infrastructure report to the NIHR on an annual basis and one of the reporting requirements is a list of research publications that have been supported by the infrastructure. Using these it is possible to trace which research publications have either been cited by NIHR infrastructure supported research publications and which research publications cite NIHR infrastructure supported publications. This helps the NIHR to understand what types of research and within its portfolio and how the research moves within the health and care ecosystem to have an impact on the real world. This is important for the NIHR to be able to demonstrate the value of publicly funded research and fulfil its mission of improving the health and wealth of the nation. In this article we describe our methods for doing this to enable other funders to be able to learn from our experience.

## Introduction

The National Institute for Health and Care Research (NIHR) is the UK’s biggest funder for health and social care research, funded by the Department of Health and Social Care (DHSC). The NIHR invests approximately £600M/year in research infrastructure ^2^, which provides a platform to enable early stage and applied health research. The NIHR infrastructure provides research expertise, specialist facilities, a research delivery workforce and support services, all of which help to support and deliver the research we fund, and research funded by others.

The NIHR’s translational research infrastructure is made up of four schemes which span the innovation pathway from early phase experimental and translational research through to clinical evaluation, implementation and adoption and spread. Our Biomedical Research Centres (BRCs) ^3^ translate discovery research into clinicial settings, our Patient Safety Research Collaborations (PSRCs, and their precursor) ^4^ evaluate patient safety, HealthTech Research Centres (HRCs, and their precursors) ^5^ evaluate health technology based interventions and our Applied Health and Social Care Collaborations (ARCs, and their precursor) ^6^ focus on implementation science and evaluations in real-world settings to help build the evidence-base for scale up and roll-out within NHS settings.

The NIHR is committed to maximising the impact of the research we support and fund ^1^ and therefore, it is crucial for the organisation to understand the mechanisms for the movement of research between these different pieces of translational research infrastructure and pathways to impact on the health and wealth of the nation. NIHR infrastructure funding can be used towards pump priming funding. However, it is largely aimed at supporting research and research delivery workforce roles which enable research to take place and career development. The research that is supported by NIHR infrastructure is typically either exclusively or partly funded by external funding. This makes attributing the impact of research projects to NIHR infrastructure difficult due to a lack of a direct, linear relationship between the infrastructure funding and specific projects in the majority of cases.

The Research Excellence Framework (REF) ^7^ is a crucial data source for impact case studies for the NIHR because it is the UK’s exercise for evaluating the quality of research produced by Higher Education Institutes (HEIs), approximately every 7 years. HEIs produce independently evidenced impact case studies which have been underpinned by excellent research from the submitting HEI. Impact case studies that do not contain commercially or otherwise sensitive information are made publicly available at the end of the assessment exercise. The last REF exercise that had been conducted at the time of writing was in 2021.

The aim of this article is to share our approach to developing an understanding of pathways to impact, enablers and barriers and lessons learnt.

## Research design and methods

### Data sources

***The REF 2021 impact case study dataset.*** The REF 2021 impact case study dataset was downloaded from the REF 2021 website ^8^. At the time of download, there were 6,361 publicly available impact case studies. Each impact case study contains information on the summary of the impacts, the underpinning research, the details of the impacts and list of publications and sources of evidence of the impacts.

***NIHR infrastructure publication data.*** Each year, NIHR infrastructure award holders submit annual reports to the NIHR. These contain information about publications that have been supported by the infrastructure. The publication data provided include a publication reference in the form of a publication citation as well as the associated Digital Object Identifiers (DOI). In some cases, Pubmed or Pubmed Central identifiers are provided instead of the DOI. Where an identifier is missing, the publication reference is used to query Pubmed to systematically source the relevant information. Publication identifiers are validated using Dimensions, Europe Pubmed Central and Pubmed.

***Linking up REF data with our publication data.*** The list of publications section of the REF 2021 impact case studies could also be optionally used by HEIs to include information about funding for the underpinning research but we found that this option was inconsistently used across the HEIs. When used, we found that this optional element typically covered project funding for which a specific amount of funding was directly attributable to the project, which is not a feature of NIHR infrastructure funding. Therefore, we used the list of publications from the REF 2021 impact case studies to link the case studies to NIHR infrastructure support.

***Additional citation analysis.*** Using forward and backward citation analysis ^9^, we also traced the movement of research as follows:

- From the BRCs to the ARCs, PSRCs and HRCs
- From the PSRCs and HRCs to the ARCs

We then ran ARC publications that had been underpinned by BRCs, PSRCs or HRCs through Overton ^10^ to search for policy citations. The policy papers were manually coded to the following NIHR priority areas of interest:

- Reducing health inequalities
- Digital health
- Artificial intelligence
- Workforce resilience

***NIHR case study development and validation.*** The REF 2021 impact case studies were coded to NIHR priority areas based on key word searches of the titles, impact summaries and details of the impact section. Case studies in our priority areas that had been supported by collaborative working across more than one infrastructure scheme were then selected for adaptation to create NIHR impact case studies. We also developed impact cases studies in the four areas of policy interest based on ‘bodies of work’ involving multi-disciplinary researchers and research teams from different parts of NIHR translational research infrastructure. Researchers or representatives of research teams were interviewed in order to understand how different pieces of NIHR infrastructure work together, enablers, barriers and challenges and evidence of impact.

***Patient and Public Involvement.*** Patients and the public were not involved in this research because it is not health research. The authors of this article work for the NIHR and have conducted this research on research to understand what is funded and supported by the NIHR, to provide transparency and accountability for the public money that the NIHR spends on research.

## Results and discussion

### NIHR infrastructure support for REF case studies

**Figure 1.**
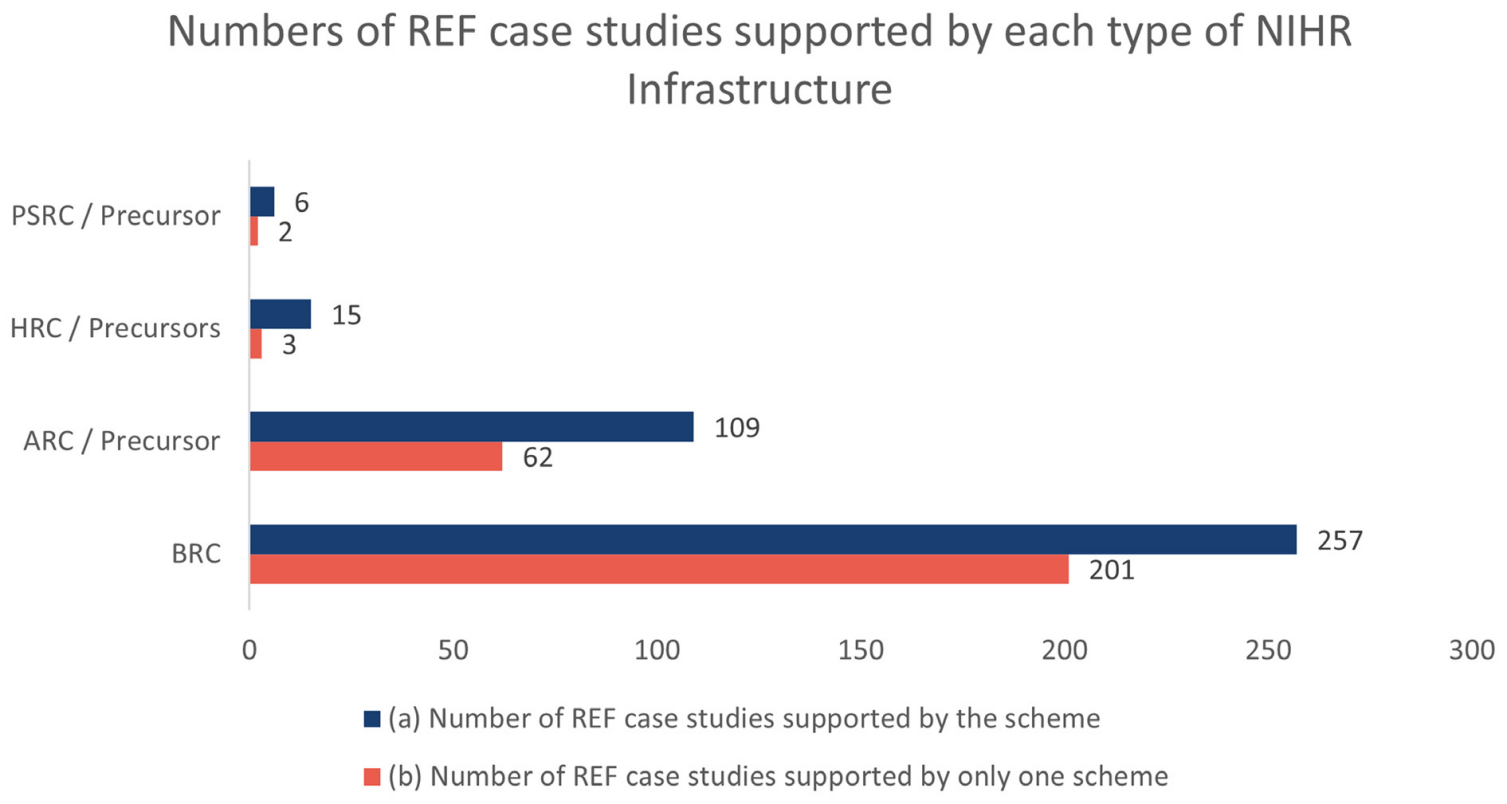
This graph shows ( **a**) how many REF impact case studies have been supported by each type of NIHR infrastructure and ( **b**) how many REF impact case studies have been supported by just one infrastructure scheme.

**Figure 2.**
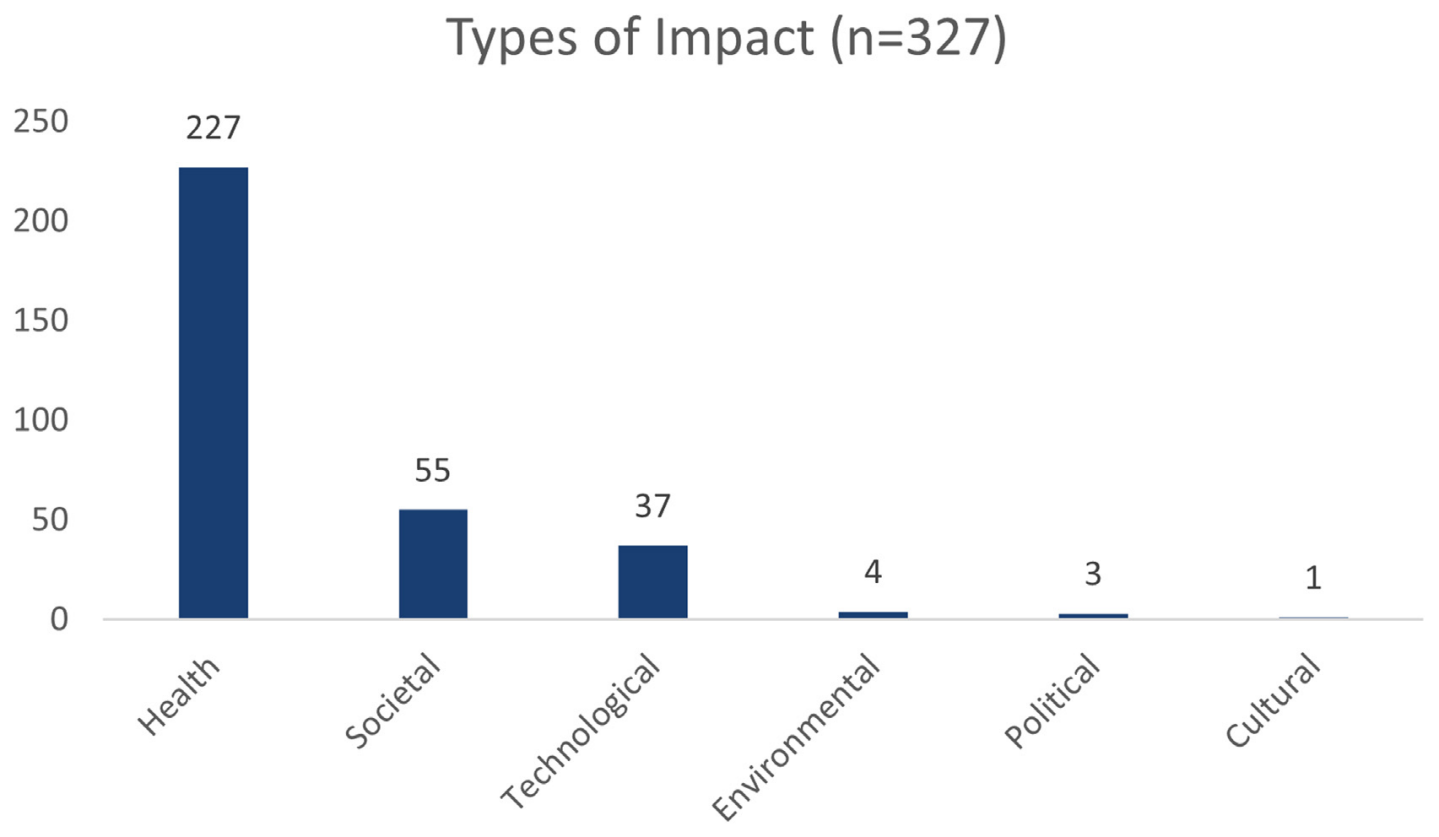
This graph shows the total number of REF impact cases that have been supported by the NIHR infrastructure, broken down by impact type.

Of the 6,361 REF impact case studies that are publicly available, the NIHR infrastructure has supported 327 of which 59 are supported by more than one scheme. Figure 1 shows the numbers for how many REF impact case studies were supported by each type of NIHR infrastructure and where impact case studies were supported by just one infrastructure scheme. Figure 2 breaks the total number of REF impact case studies supported by NIHR infrastructure i.e 327 into the different types of impact, as classified by the REF dataset.

### Insights from researcher interviews

The interviews that formed part of the case study development process explored how different pieces of NIHR infrastructure work together, enablers and barriers for impact. This revealed five areas of strength in terms of how NIHR infrastructure delivers impact and areas for future work to better understand some of these mechanisms.

The first is strategic partnerships and collaborations between different pieces of NIHR infrastructure and how these help to move projects down the innovation pathway ^11^. An example of this is a strategic partnership between the MindTech HRC ^12^ and the SLaM BRC ^13^. The BRC brings expertise in the early stage investigation of mechancisms that underpin new interventions or technologies. The HRC brings expertise in the evaluation of these interventions or technologies in NHS settings. By working together, these two pieces of infrastructure can ensure that minimal time and money is lost in developing products that won’t work in the NHS. This way of working is also called ‘fast failure’ ^14^ and enables the movement of interventions from bench to bedside quicker than the typical 10–15 year time lag between research and impact ^15^.

The ability to embed research in the NHS is a key strength of NIHR infrastructure. An example of this is research ^16^ by the Yorkshire and Humbar PSRC which looked at the effects of wellbeing and burnout of healthcare professionals on patient safety. Prior to this research, the interest in NHS workforce resilience was primarily on retention and skills rather than wellbeing. The direct involvement of clinical and academic staff in research projects also means that clinical data that are not routinely collected can be monitored to help understand unmet needs but also build the evidence base for novel interventions.

The case study development process revealed close working relationships between the NIHR infrastructure, particulary the ARCs, and commissioners through the Health Innovation Networks (HINs) ^17^. This allows NIHR infrastructure to build the evidence base for and push new interventions and technologies into the NHS but also pull ideas and interventions from the HINs to evaluate in various ways. An example of this is ARC North Thames which evaluated the benefits of co-locating welfare advice services in GP settings, demonstrating significant improvements in patient mental health and well-being, reaching those most in need and supporting healthcare staff. This directly led to services being retained in primary care settings in specific parts of England and roll-out nationally in Scotland ^18^. Understanding the push/pull mechanisms between the NIHR infrastructure and the HINs and wider health and care system is an area for future work.

The next area of strength is core funding. The infrastructure provides research expertise, specialist facilities, a research delivery workforce and support services, all of which help to support and deliver the research we fund, and research funded by others ^2^. This means that infrastructure centres have multi-disciplinary expertise and well developed patient and public involvement and engagement (PPIE) ^19^ functions which enable early and seamless input from a varierty of experts based on need. In the absence of these, external funding would need to be sought for specific projects which could administrative delays or lead to the ‘death’ of projects considered high risk or not in line with specific funder priorities. The infrastructure also enables sharing and linking with various databases and sources which enriches the research. Another area for future work is understanding why this ‘valley of death’ ^20^ exists for projects and what additional support can be provided to help.

The NIHR infrastructure supports PhD students, post-docs and other researchers from a range of disciplines and at different career stages, recruited to support specific projects. Core funding enables a greatly reduced reliance on external, short-term, precarious funding ^21^ to attract and retain research talent. The buy-out of their time allows them to develop and explore new ideas, sometimes using seed funding from the infrastructure, to get them to a stage where external funding can be sought. Early career researchers also have opportunities to co-lead various initiatives in the PPIE or knowledge mobilistion space, for example, which helps with career development for succession planning within the infrastructure. The NIHR Academy ^22^ provides training and support to health and care researchers at all stages of their careers to build national health and care research capacity and capability. Evaluating our capacity building programmes helps us to understand their value and further develop them to meet the dynamic needs of our workforce.

The interviews also revealed a specific need for more join up across the social care system which is fragmented due to the fact that is it made up of several private organisations, commissioned to deliver NHS or community care services. This poses a challenge for adoption, implementation and downstream impacts on patients, due to the amount of time and costs of working with multiple stakeholders across the care system. Targeted funding to support researchers with policy- or implementation-ready research findings to work with multiple companies within the care system could help with this.

## Conclusions

Publications are typically used to track what’s in a funding portfolio in terms of research areas but also reach of research through various altmetrics. The use of forward and backward citation analysis can also help research funders to understand how research is moving between different parts of their funding portfolios, pathways to impact and any gaps and opportunities. However, this does come with some challenges around data quality and timeliness, for example, which need mitigation.

## Data Availability

The data are publicly available: REF 2021 impact case studies ^8^. NIHR impact case studies ^23^. Research publications can be accessed using databases such as Pubmed or Europe PMC. We have used Dimensions and Overton for altmetric analyses.
