# Supplementary material for: Gaining insights into a funding portfolio through publication tracking
Source: NIHR Open Res. 2025 Dec 1;5:62. Originally published 2025 Jul 22. [Version 3] doi: 10.3310/nihropenres.14022.3 (PMC12627934; doi:10.3310/nihropenres.14022.3)
Supplement: Peer review history

# Reviewer report: Reviewer response for version 3

**DOI:** 10.3310/nihropenres.15436.r38755

**Published:** 2026-01-07

**Authors:** Rachel Heyard

**Affiliations:** 1 University of Zurich, Zurich, Switzerland

The paper by Suri-Ogilvie presents an interesting analysis on the actual impact of the research infrastructure funded by NIHR. By linking data from the REF 2021 and data/information on publications of research supported by NIHR infrastucture (even if externally funded) the authors bring an innovative / novel perspective to the impact of infrastructure funding. The linked dataset was then augmented via a foreward-backward citation search and a search for citations in policy documents (through Overton). On top of this already very interesting data and quantititative analysis, the authors also conducted interviews to better understand links between infrastructure funding and impact beyond publications. 

 While the data collected and linked by the authors is extremely rich and has great potential, the presented manuscript lacks transparency in crucial aspects of the  methodology and the presentation of the results.

 First of all, the semi-structured interviews, which seem to be crucial for the development of the case studies and take a center stage in the results are first mentioned on page 5, at the very end of the methods. There is no mention of interviews in the abstract or title or the data sources. Interview transcripts etc should in my opinion be seen as (qualitative) data. Specifically with respect to the interviews that were conducted the manuscript is lacking transparency. I'd recommend the authors have a look at a reporting guideline for research based on interviews (e.g., CREDES). How were researchers or representatives selected or found? Are these authors of the publications included in the bibliographic analysis, or other? How many interviews with how many researchers were conducted? Were they online or onsite? Who conducted the interviews? Was there an interview guide? Were interviews piloted? ETC. Further, a table or so with some descriptive statistics on both interviewers and interviewees is needed (e.g., ECRs or established researchers? disciplines represented? ...)

 Also for the bibliographic data analysis I am missing transparency - I personally would not be able to reproduce the analysis. For example, the authors need to provide dates of access of the various datasets, since they seem to be "living", i.e., updated regularly. For the backward and forward citation, it should say in the methods that the search was done within Dimensions; and I would have like to find the list of initial DOIs somewhere, and again importantly the date of the search - which would today already give different results for the forward part. The same goes for Overton, possibly also the search string that was used. For the keyword search conducted on titles etc of the REF impact case studies, I would like to see the whole list of keywords somewhere (if it is long, in an appendix) and not just a selection. 

 As for the presentation of the results, the discussion of the quantitative analysis is very limited. I would have expected some sort of plot / diagram showing the linkage of the different data sources (maybe a sankey plot or similar?). Figure 2, for me, is non sensical - what data is represented here? It is not the 327 case studies, right? since then, the numbers do not add up. I could not find any mention of the policy paper analysis in the results - or at least I could not understand how they contributed to the results. 

 The discussion then continues with a long presentation of case studies - but it is unclear how they came together from the presented methodology. It therefore reads a bit like cherry picked stories and henceforth not very trustworth.

 All in all, I find that the data and the idea presented by the authors have a lot of potential - I however am less convinced with the execution, mainly because the quality of the reporting and the transparency leave lots of open questions. 

Is the work clearly and accurately presented and does it cite the current literature?

Partly

If applicable, is the statistical analysis and its interpretation appropriate?

Not applicable

Are all the source data underlying the results available to ensure full reproducibility?

No

Is the study design appropriate and is the work technically sound?

No

Are the conclusions drawn adequately supported by the results?

Partly

Are sufficient details of methods and analysis provided to allow replication by others?

No

Reviewer Expertise:

Statistics; funding allocation systems; meta-research; reproducibility; open science

I confirm that I have read this submission and believe that I have an appropriate level of expertise to state that I do not consider it to be of an acceptable scientific standard, for reasons outlined above.

## References

[1] : Consolidated criteria for reporting qualitative research (COREQ): a 32-item checklist for interviews and focus groups. *International Journal for Quality in Health Care* .2007;19(6) : 10.1093/intqhc/mzm042 349-357 10.1093/intqhc/mzm042 17872937

# Reviewer report: Reviewer response for version 3

**DOI:** 10.3310/nihropenres.15436.r38606

**Published:** 2026-01-07

**Authors:** Erik Canton

**Affiliations:** 1 Directorate-General for Research and Innovation, European Commission, Brussels, Belgium

The authors have improved the manuscript based on the feedback provided. The authors did not provide a more elaborate discussion on the forward and backward citation analysis in the revised version. If this information is available through other means I would encourage the authors to include a reference so that interested readers can understand the results of the analysis more in-depth.

Is the work clearly and accurately presented and does it cite the current literature?

Partly

If applicable, is the statistical analysis and its interpretation appropriate?

Partly

Are all the source data underlying the results available to ensure full reproducibility?

Partly

Is the study design appropriate and is the work technically sound?

Partly

Are the conclusions drawn adequately supported by the results?

Partly

Are sufficient details of methods and analysis provided to allow replication by others?

Partly

Reviewer Expertise:

NA

I confirm that I have read this submission and believe that I have an appropriate level of expertise to confirm that it is of an acceptable scientific standard.

# Reviewer report: Reviewer response for version 3

**DOI:** 10.3310/nihropenres.15436.r38756

**Published:** 2026-01-07

**Authors:** Carter Bloch

**Affiliations:** 1 Department of Political Science, Aarhus University, Aarhus, Central Denmark Region, Denmark

This paper is clear and provides and interesting description of the NIHR's approach to study impact through the linking of different grants. I can also see that the authors have addressed comments to previous versions of this paper. The use of examples of cases help to illustrate what can be gained from this method. 

 One point that might be worth further consideration and clarification is the "forward and backward citation analysis". There still seems to be some uncertainty of what this entails. For example is it mainly just used to link grants (as mentioned in the manuscript), or are these forward and backward citations used in greater detail to aid in understanding how subsequent grant work (as described in the citing paper) was based on the previous research?

Is the work clearly and accurately presented and does it cite the current literature?

Yes

If applicable, is the statistical analysis and its interpretation appropriate?

Not applicable

Are all the source data underlying the results available to ensure full reproducibility?

Yes

Is the study design appropriate and is the work technically sound?

Yes

Are the conclusions drawn adequately supported by the results?

Yes

Are sufficient details of methods and analysis provided to allow replication by others?

Partly

Reviewer Expertise:

Research funding evaluation, analysis of societal impact of research

I confirm that I have read this submission and believe that I have an appropriate level of expertise to confirm that it is of an acceptable scientific standard.

# Reviewer report: Reviewer response for version 3

**DOI:** 10.3310/nihropenres.15436.r38607

**Published:** 2025-12-23

**Authors:** Shaoxiong Brian Xu

**Affiliations:** 1 The Hong Kong Polytechnic University, Hong Kong, China

The revised manuscript contains no mention of retracted publications. If the study did not involve retracted publications, it should be explicitly stated in the methods section, and a one-sentence mention would do. If the study did involve retracted publications, how they were handled should be disclosed.

Is the work clearly and accurately presented and does it cite the current literature?

Yes

If applicable, is the statistical analysis and its interpretation appropriate?

Yes

Are all the source data underlying the results available to ensure full reproducibility?

Yes

Is the study design appropriate and is the work technically sound?

Partly

Are the conclusions drawn adequately supported by the results?

Partly

Are sufficient details of methods and analysis provided to allow replication by others?

Partly

Reviewer Expertise:

Retraction; Research integrity

I confirm that I have read this submission and believe that I have an appropriate level of expertise to confirm that it is of an acceptable scientific standard, however I have significant reservations, as outlined above.

# Reviewer report: Reviewer response for version 2

**DOI:** 10.3310/nihropenres.15363.r37657

**Published:** 2025-11-18

**Authors:** Shaoxiong Brian Xu

**Affiliations:** 1 The Hong Kong Polytechnic University, Hong Kong, China

The authors have substantially improved the manuscript by incorporating feedback from previous reviewers, resulting in a fairly solid manuscript. However, one important methodological and analytical considerations require attention.

 As a retraction researcher, I have a fundamental concern regarding the handling of retracted publications in the citation analysis. The manuscript does not specify whether retracted publications were included in the forward and backward citation analyses or how they were treated if encountered. Given the documented high-stakes nature of retracted research in medical and life sciences and evidence of continued post-retraction citations, any retracted publications should be explicitly excluded from the citation analysis to ensure accurate assessment of genuine research impact.

 I recommend conducting a focused analysis of excluded retracted publications, if any, that received NIHR funding or infrastructure support. This analysis would identify high-risk research areas and researchers requiring targeted research integrity interventions, providing valuable insights for the NIHR's quality assurance and oversight functions.

 Three specific issues need correction. First, the final sentence of the first paragraph in "Research design and methods" mentions "sources of evidence of the impacts" without adequate explanation - a brief introduction to these sources would enhance clarity. Second, the heading "our publication data" in the third paragraph of the same section lacks specificity and could be improved by changing it to "Linking up REF data with NIHR data" for better signposting. Third, the first sentence of the final paragraph in "Results and discussion" contains a grammatical error where "that is it made up of" should be corrected to "that it is made up of".

Is the work clearly and accurately presented and does it cite the current literature?

Yes

If applicable, is the statistical analysis and its interpretation appropriate?

Yes

Are all the source data underlying the results available to ensure full reproducibility?

Yes

Is the study design appropriate and is the work technically sound?

Partly

Are the conclusions drawn adequately supported by the results?

Partly

Are sufficient details of methods and analysis provided to allow replication by others?

Partly

Reviewer Expertise:

Retraction; Research integrity

I confirm that I have read this submission and believe that I have an appropriate level of expertise to confirm that it is of an acceptable scientific standard, however I have significant reservations, as outlined above.

# Response

**Published:** 2025-11-27

**Authors:** Reetika Suri-Ogilvie

**Affiliations:** Not Applicable, UK

We thank our reviewer for the time taken to read and comment on our manuscript and their kind and constructive feedback. We have fixed the minor typos that have been pointed out.

 While retracted publications are a very important consideration for publication analysis, taking a specific approach to analysing them was out of scope for this work. However, they will certainly be a consideration for future work. For this work, we took an annual data cut of papers that held the status of ‘published’ at the time of the data cut and took those papers forward into our downstream analysis. We hope that this response provides clarity on our approach.

# Reviewer report: Reviewer response for version 2

**DOI:** 10.3310/nihropenres.15363.r37563

**Published:** 2025-10-31

**Authors:** Josie Coburn

**Affiliations:** 1 SPRU - Science Policy Research Unit, University of Sussex, Brighton, England, UK

Thank you for updating this article. I think the clarity has improved and I'm happy with the quality.

 I think one more minor typo has been introduced in the editing in the introduction: "from early phase experimental and research through to clinical evaluation" - I would suggest removing the "and" here.

Is the work clearly and accurately presented and does it cite the current literature?

Partly

If applicable, is the statistical analysis and its interpretation appropriate?

Not applicable

Are all the source data underlying the results available to ensure full reproducibility?

Partly

Is the study design appropriate and is the work technically sound?

Partly

Are the conclusions drawn adequately supported by the results?

Yes

Are sufficient details of methods and analysis provided to allow replication by others?

Partly

Reviewer Expertise:

Science, technology and innovation policy; science and technology studies; research on research; metascience; research targeting; research funding; research evaluation; research impact

I confirm that I have read this submission and believe that I have an appropriate level of expertise to confirm that it is of an acceptable scientific standard.

# Reviewer report: Reviewer response for version 1

**DOI:** 10.3310/nihropenres.15250.r36488

**Published:** 2025-09-03

**Authors:** Josie Coburn

**Affiliations:** 1 SPRU - Science Policy Research Unit, University of Sussex, Brighton, England, UK

This paper provides a very interesting investigation of the impacts of UK National Institute for Health and Care Research (NIHR) infrastructure funding by linking publications cited by UK Research Excellence Framework (REF) impact case studies with those supported by NIHR infrastructure funding, including which scheme(s) they were funded by; by tracing forwards and backwards citations associated with these publications; by identifying policy papers that cite these papers; by classifying them according to NIHR priority areas; and by developing NIHR impact case studies.

 I have a few suggestions to improve the clarity of the paper for readers who may not be familiar with some elements of the research, and to provide more details about the methods and results.

 In the introduction, the NIHR translational research infrastructure and four schemes are introduced. I would suggest adding a little more detail about how the translational research infrastructure relates to the (broader?) NIHR infrastructure, and about the four schemes introduced in paragraph 2 of the introduction.

 In the methods section, in the 'Additional citation analysis section', I would suggest adding more details about the forwards and backwards citation analysis, in particular with reference to the four schemes and how they relate to each other. This research involves REF impact case studies, NIHR infrastructure publications, forwards and backwards citation analysis, the four NIHR translational research schemes, and policy papers from Overton, which are then coded to NIHR priority areas of interest. I think I diagram providing more details about how these different entities relate to each other might help to improve the clarity of the data and methods section. Some more specific questions that could be clarified are: Where are the NIHR priority areas of interest from (bottom of page 4 and also reference number 23, which leads to a broader 'What we do' page and not to case studies)? Which key words were used for the key word searches and how were they identified (top of page 5)? Why were case studies selected that had been supported by more than one infrastructure scheme and not only one? What were the 'bodies of work' highlighted on page 5 and how were they used to develop impact case studies? Where are the NIHR case studies and how were they validated? What sort of interviews were conducted, for how long, with whom and how was the qualitative data analysed?

 In the results section, I would love to see more descriptive statistics about the data. For instance, how many publications were linked, how many links forwards and backwards were there between the different types of data, how many policy documents were linked, how many policy documents were assigned to each priority area? Can you say anything about the topics of the NIHR infrastructure grants, publications and policy documents compared to the topics of the case studies? Are they the same or different?

 The insights from research interviews are organised into 5 areas of strength and areas of future work. In the third area, Health Innovation Networks (HINs) are introduced, but not defined. I would suggest adding a little more detail about what HINs are and what they do. I find areas 3 and 1 to be quite similar. Please consider clarifying the difference between these two areas.

 There are also just a few minor typos, e.g. page 2: Plain English Summary – there are a few typos in this text and the clarity of it could be improved; page 5: Humbar = Humber; page 6: "In the absence of these, external funding would need to be sought for specific projects which could * administrative delays…", I think there is a word missing where I have inserted the *.

 Overall, I think this is an interesting and valuable paper. I would just like to see more details.

 This paper, which links funding proposals and REF impact case studies, may be of interest to you: Yaqub O et al 2023 (Ref 1)

Is the work clearly and accurately presented and does it cite the current literature?

Partly

If applicable, is the statistical analysis and its interpretation appropriate?

Not applicable

Are all the source data underlying the results available to ensure full reproducibility?

Partly

Is the study design appropriate and is the work technically sound?

Partly

Are the conclusions drawn adequately supported by the results?

Yes

Are sufficient details of methods and analysis provided to allow replication by others?

Partly

Reviewer Expertise:

Science, technology and innovation policy; science and technology studies; research on research; metascience; research targeting; research funding; research evaluation; research impact

I confirm that I have read this submission and believe that I have an appropriate level of expertise to confirm that it is of an acceptable scientific standard, however I have significant reservations, as outlined above.

## References

[1] : How unpredictable is research impact? Evidence from the UK’s Research Excellence Framework. *Research Evaluation* .2023;32(2) : 10.1093/reseval/rvad019 273-285 10.1093/reseval/rvad019

# Response

**Published:** 2025-09-26

**Authors:** Reetika Suri-Ogilvie

**Affiliations:** Not Applicable, UK

We would like to thank the reviewer for their time taken to provide feedback on our manuscript and for their positive and constructive comments.

 We have added a bit more detail about each of our infrastructure schemes and how the research infrastructure differs from our research delivery infrastructure. We have also included a flow diagram to help with reader understanding of how we have thought about the movement of research within our research infrastructure portfolio.

 We have rectified reference 23.

 We have provided a bit more detail about the key word searches but we found these of limited utility due to the vagueness of the themes we were searching for, so the majority of the filtering was done manually, as has now been explained in the text.

 We have provided an explanation of the term ‘bodies of work’ in the text. We have also explained why case studies where different pieces of infrastructure had worked together were of interest to us and provided a bit more detail about the interviews we conducted and analysed.

 We have not included descriptive statistics for the citation analysis in this manuscript as we would like the focus of this manuscript to remain on pathways and mechanisms for impact and any gaps. With the citation analysis, we are simply showcasing a use case for tracing the movement of research. The NIHR has a commitment to the responsible use of metrics, and as such, we would not like to focus on numbers that were not meaningful for this piece of work because the focus of this manuscript is not on what ‘good’ should or might look like in terms of citations but on understanding how we might be able to use citations to follow the movement of research within our portfolio and towards impact.  

 In the discussion, we have provided a bit more information about the HINs and have clarified the difference between strength 1 and 3. We appreciate that this was previously not as clearly articulated as we would have liked.

 We are undertaking a piece of work to look at clusters of research activity and expertise within our infrastructure portfolio. However, it is very difficult to directly compare project topics with case studies. There are a number of reasons for this. The NIHR infrastructure portfolio is made up of approximately 16,000 projects in any given financial year. These are typically at different stages of maturity in terms of impact. Additionally, we have found that the majority of successful impact examples come from ‘bodies of work’ which involve multi-disciplinary expertise and academics from different parts of infrastructure working with other stakeholders from across the health and care ecosystem, rather than individual projects. Furthermore, research can have unintended impacts, which, in some cases, the researchers are completely unaware of or have had no involvement with or influence over. A list of NIHR infrastructure supported projects can be found here.

# Reviewer report: Reviewer response for version 1

**DOI:** 10.3310/nihropenres.15250.r36489

**Published:** 2025-08-23

**Authors:** Erik Canton

**Affiliations:** 1 Directorate-General for Research and Innovation, European Commission, Brussels, Belgium

This paper by Suri-Ogilvie et al. on "Gaining insights into a funding portfolio through publication tracking" investigates the impact of the research supported and funded by the National Institute for Health and Care (NIHR) in the UK. To maximise the impact of the research, the authors want to better understand the movement of research projects between the different pieces of translational research infrastructure and pathways. The authors use publication analysis of the impact case studies and conclude that forward and backward citation analysis of publications help funders understand how research is moving between different parts of their funding portfolios.

 The authors have chosen an important and interesting topic with direct policy relevance. I have a number of comments that could help the authors to further develop the analysis for the article and maybe also in follow-up research.

 First, as a general comment, the readability of the article can be improved. For readers not very familiar with the NIHR the text is sometimes difficult to follow, also because of the use of many acronyms and the sometimes very short explanatory text related to NIHR's organisation and functioning. Also, there are several typos and grammatical issues in the text that should be fixed (e.g. "mobilistion").

 Second, there are some descriptive statistics and figures in the article which are interesting, but they could benefit from some further elaboration. Figure 1 shows how many impact case studies have been supported by the different types of NIHR infrastructure, whereas figure 2 shows the number of impact cases broken down by impact type. It would be interesting to see some more reflection on this figure 2, for example on efficiency/effectiveness of the research vis-à-vis these different impact types and on the robustness of the methodologies employed in these studies to infer causality. From the abstract and introduction I was also expecting some insights from the citation analysis, on backward and forward citations and how these citations help to move between the different pieces of research infrastructure. The publication tracking part mentioned in the manuscript's title should be further developed in the text, and the reader will be curious to learn more about this.

 Third, the authors do not provide a clear definition of funding portfolio and how portfolio considerations can help improve the impact of the funded research. Do they have in mind some type of ex ante portfolio model as in Canton, 2025 (Ref 1) with implications for project financing decisions (ex ante, so before starting the project) or do the authors mostly refer to an ex post approach, where the value is maximised of the ongoing set of projects within a portfolio? It seems that the authors have the latter approach in mind, but it might be interesting to also include some reflections in the article on financing decisions when portfolio considerations play a role. The authors seem to be interested in achieving societal value by promoting projects to move more quickly from low TRLs to higher ones, as covered by the NIHR, but they also refer to different impact types and it is not clear to me which mechanisms the authors want to emphasise, and how this relates to the citation analysis.

 More generally, and also referring to figure 2 in the article, the analysis leaves unanswered the question whether additional considerations, such as impact type, should matter for funding decisions, in addition to more traditional criteria such as academic excellence. It would be interesting to include some reflections on that in the article. In particular, can the impact of the funded projects be further increased by including portfolio considerations in the financing decisions of the projects, deviating from the more traditional merit-based approach?

Is the work clearly and accurately presented and does it cite the current literature?

Partly

If applicable, is the statistical analysis and its interpretation appropriate?

Partly

Are all the source data underlying the results available to ensure full reproducibility?

Partly

Is the study design appropriate and is the work technically sound?

Partly

Are the conclusions drawn adequately supported by the results?

Partly

Are sufficient details of methods and analysis provided to allow replication by others?

Partly

Reviewer Expertise:

Economics of research and innovation

I confirm that I have read this submission and believe that I have an appropriate level of expertise to confirm that it is of an acceptable scientific standard, however I have significant reservations, as outlined above.

## References

[1] : A portfolio approach to research funding. *Research Policy* .2025;54(1) : 10.1016/j.respol.2024.105129 10.1016/j.respol.2024.105129

# Response

**Published:** 2025-09-26

**Authors:** Reetika Suri-Ogilvie

**Affiliations:** Not Applicable, UK

We thank our reviewers for their time taken to review our work and kind and helpful comments.

 We have fixed the typos in the manuscript. We have also included a bit more detail about the different types of NIHR infrastructure. However, it is a complex funded portfolio, and we did not want to include too much detail which deviates from the methodology and its use that we would like to focus on in this article. Further details of each piece of NIHR infrastructure can be found in the reference links.

 The Research Excellence Framework (REF) is an exercise that is conduced by Research England, which is a completely separate entity from the NIHR. Participation in the REF is at a university level whereas the majority of NIHR infrastructure is hosted by NHS Trusts which are partnered with UK universities. REF impact case studies are presented from a university’s perspective and represent only a small proportion of carefully selected and curated research being undertaken in any given university. This, combined with the fact that NIHR infrastructure enables and facilitates, through expertise, collaborations and networks, but may not directly or fully fund, research, makes it very difficult to define a linear relationship between NIHR infrastructure supported research and impact. We used citation analysis as a starting point to determine where NIHR infrastructure has played an enabling role in research, and qualitative methods to further understand the pathways to impact. Pathways to impact can be convoluted and messy and impacts can be unintended which also makes it difficult to comment on the relationship between research methodologies and efficiency of research and resulting impacts.

 We have a provided a bit more detail on our thinking around forward and backward citation analysis and how it has helped us to understand the movement of research between different parts of our funded portfolio. It would be helpful to understand what further insights might be of interest to our readers.

 The aim of this article is to present our methodologies and learning from using citation analysis to understand pathways to impact through the movement of research within our infrastructure portfolio. The NIHR spends public money on research, and therefore, showing return on investment and being accountable for this spend is crucial. Lessons learnt from this work have fed into thinking about gaps and opportunities for our infrastructure to enable impact at a systems level, rather than funding decisions on individual projects based on impact types. Additionally, NIHR infrastructure is funded to be responsive to policy needs and the needs of the health and care system, for which impact might look like a variety of different things that deviate from traditional impact types.
